# Evaluation of Astigmatic Correction Using Vector Analysis after Combined Femtosecond Laser-Assisted Phacoemulsification and Intrastromal Arcuate Keratotomy

**DOI:** 10.1155/2021/2860840

**Published:** 2021-01-29

**Authors:** Su Young Moon, Ho Seok Chung, Jae Hyuck Lee, So Young Park, Hun Lee, Jae Young Kim, Hungwon Tchah

**Affiliations:** ^1^Department of Ophthalmology, Asan Medical Center, University of Ulsan College of Medicine, Seoul 05505, Republic of Korea; ^2^Department of Ophthalmology, Dankook University Hospital, Dankook University College of Medicine, Cheonan 31116, Republic of Korea

## Abstract

The aim of this study was to evaluate astigmatic correction in patients with mild to moderate astigmatism after combined femtosecond laser-assisted cataract surgery (FLACS) and intrastromal arcuate keratotomy (ISAK), using vector analysis. This retrospective study included patients with corneal astigmatism of 0.5–3.0 diopters (D) who underwent FLACS and ISAK. Vector analyses of astigmatism were performed using the Alpins method, considering three vectors: target-induced astigmatism (TIA), surgically induced astigmatism (SIA), and difference vector (DV). Magnitude of error (ME), angle of error (AE), correction index (CI), and coefficient of adjustment (CA) were calculated. Subgroup analysis according to the axis of astigmatism, patient age, and white to white (WTW) diameter was conducted. In total, for the 79 eyes of 79 patients, the TIA was 1.21 ± 0.52 *D*, the SIA was 0.76 ± 0.53 D, and the DV was 0.86 ± 0.50 D. The ME (difference between SIA and TIA) was −0.46 ± 0.45 D, and the CI (ratio of SIA and TIA) was 0.62 ± 0.34; both these parameters demonstrated slight undercorrection. The CA (inverse of the CI) was 2.48 ± 2.61. The AE was 4.02° ± 28.7°, and the absolute AE was 21.7° ± 19.0°. In the univariate regression analyses to identify factors that affected the CI, there was a negative correlation between age and the CI (*P*=0.022). In conclusion, vector analysis after the combined FLACS and ISAK revealed slight undercorrection, regardless of the astigmatism meridian. The precision of the nomogram should be improved through long-term vector analysis for the results of arcuate keratotomy and through further research on the relationship between patient demographics and CI. Overall, this study has shown that FLACS and ISAK could reduce postoperative corneal astigmatism effectively and safely.

## 1. Introduction

Although modern cataract surgery allows for rapid visual recovery, preexisting corneal astigmatism remains a common obstacle to achieving excellent uncorrected visual acuity. Uncorrected astigmatism significantly compromises a patient's vision and leads to reduced quality of life [1]. Corneal astigmatism greater than 1.0 diopter (*D*) was found in 27.95% of 23,239 eyes in a recent study [2]. A variety of treatment options exist to reduce this corneal astigmatism during surgery, including toric intraocular lens (IOL) implantation, opposite clear corneal incisions, manual arcuate keratotomy, limbal relaxing incision, or a combination of these approaches [3–6].

The advent of femtosecond laser technology allows precise control of corneal incisions, capsulorhexis (centration, size, and regularity), and nucleus fragmentation and may yield promising surgical outcomes and early recovery of visual acuity of patients, with effects on IOL centration and reduced usage of total phacoemulsification energy. Among these surgical steps and as compared to manually delivered keratotomy, arcuate keratotomy (AK) with femtosecond laser has been known to be efficacious in reducing corneal astigmatism in mild to moderate corneal astigmatism, with high reliability and reproducibility [7–9]. According to a recent study, the outcomes of AK with femtosecond laser were comparable to those of toric IOL implantation in eyes with low to moderate astigmatism [10].

The position, length, and depth of the incisions are guided by nomograms based on the amount of astigmatism, type of astigmatism, and the patient's age. There are various types of nomograms according to the laser devices and types of AK—paired or single type and penetrating or intrastromal AK, respectively [11–14]. In intrastromal AK (ISAK), the cut is performed within the stroma and does not reach Bowman's layer. The ISAK has the advantage of theoretical elimination of the risk of infection and minimization of postoperative pain. In addition, wound gape and epithelial ingrowth could be avoided because of the absence of an open wound [15]. The mainly used nomogram for ISAK is Version 3 nomogram provided by Dr. Julian Stevens in 2015 [16]. However, studies on the evaluation of nomograms are limited.

The aim of our study was to evaluate astigmatic correction in patients with mild to moderate astigmatism using Alpins vector analysis method after combined femtosecond laser-assisted cataract surgery (FLACS) and ISAK using a nomogram provided by Dr. Julian Stevens. Additionally, we investigated an approach to increase the accuracy of ISAK and to determine the adjustments needed for the nomogram.

## 2. Subjects and Methods

This retrospective study included patients who underwent combined FLACS and ISAK between 2016 and 2019 at the Asan Medical Center in Seoul, Korea. All procedures adhered to the tenets of the Declaration of Helsinki, and the study was approved by the Institutional Review Board of Asan Medical Center at the University of Ulsan in Seoul, Korea.

### 2.1. Preoperative and Postoperative Examinations

A standard preoperative ophthalmic examination that included Autorefractive keratometer (KR-1; Topcon Corp., Japan), noncontact tonometry (CT-80; Topcon Corp., Japan), slit-lamp biomicroscopy, fundoscopy, partial coherence interferometry (IOLMaster 500; Carl Zeiss Meditec AG, Germany), specular microscopy (Cellchek SL; Konan Medical USA, Inc., CA, USA), scanning-slit topography (ORBscan; Bausch and Lomb, Inc., NY, USA), and ocular aberrometry (OPD-Scan; Nidek Co., Ltd., Japan) was performed. Preoperative ocular biometric measurements, including axial length, anterior chamber depth, and keratometric values, were performed to calculate the IOL power using partial coherence interferometry. Steep keratometry (K), flat K, and steep meridian values to be entered into the calculator were determined by the surgeon, with consideration of data obtained with the autorefractive keratometer, simulated K of scanning-slit topography, and partial coherence interferometry. Patients were evaluated at one day, one week, one month, and three months after surgery. The visual acuity, intraocular pressure, and keratometric measurements through the autokeratometer were assessed every visit. The manifest refraction and keratometric measurement obtained through scanning-slit topography were assessed at one month and three months after surgery.

### 2.2. Surgical Techniques

A single experienced surgeon performed FLACS using a Catalys Precision Laser System (Abbott Medical Optics, Inc., CA, USA). The central corneal thickness (CCT) and longest white-to-white (WTW) diameter were obtained during surgery on the laser platform. To avoid the effect of cyclotorsion in the supine position, the horizontal line was marked on the eye with a pen while the patient was sitting. The horizontal line was then aligned on the laser's nonapplanating liquid optics interface. A femtosecond laser was used to perform capsulorhexis capsulotomy and lens fragmentation, followed by ISAK when the corneal astigmatism exceeded 0.50 D and was not exceeding 3.0 D. The length of the arcuate keratotomy was determined using the nomogram provided by Dr. Julian Stevens [16].

All ISAKs consisted of a pair of symmetric intrastromal incisions with a depth of 20–80% of the corneal thickness and a limbus-based flap, 8.0 mm in diameter. The arc length ranged from 30° to 90°, depending on the patient's age and the magnitude and axis of astigmatism. After the laser procedure, a main limbal incision was created using a 2.2 mm keratome, and the anterior capsule button was removed using forceps.

Phacoemulsification was performed using a Whitestar Signature Phacoemulsification System (Abbott Medical Optics, Inc., CA, USA). A single-piece IOL (Tecnis ZCB00; Abbott Medical Optics, Inc., CA, USA) was implanted in all eyes. Postoperatively, all patients were administered 1% prednisolone acetate suspension (Pred Forte®; Allergan, Inc., CA, USA) and 0.5% moxifloxacin (Vigamox®; Alcon Laboratories, TX, USA) for one month.

### 2.3. Analysis of Astigmatic Correction

The magnitude and axis of the keratometric astigmatism were used to determine the ISAK profiles and to analyze the postoperative outcome of the astigmatic correction. Vector analysis and graphic displays were performed using the Alpins method, facilitated by the ASSORT Group Analysis Calculator [17].

In this study, target-induced astigmatism (TIA) was defined as the astigmatic change the surgeon intended to induce to correct the patient's preexisting astigmatism based on the magnitude and axis. The astigmatic correction was calculated using a nomogram calculator that incorporated the value of keratometric astigmatism (magnitude and axis) and the surgeon-specific flattening effect (−0.25 D to 0.00 D) of a 2.2 mm main incision and a 1.0 mm side port. The surgically induced astigmatism (SIA) is the amount and axis of a stigmatic change achieved by the ISAK. The difference vector (DV) is the astigmatic change that would enable the initial surgery to achieve its intended target based on the magnitude and axis. The DV is an absolute measure of success and is preferably zero.

The arithmetic difference values between the SIA and TIA magnitudes and axes were defined as the magnitude of error (ME) and angle of error (AE), respectively. The ME is positive for overcorrections and negative for undercorrection. The AE is positive if the achieved correction is on an axis counterclockwise to where it was intended and negative if the achieved correction is clockwise to its intended axis. The correction index (CI), coefficient of adjustment (CA), and flattening index (FI) were also calculated. The CI is the ratio of the SIA to the TIA, with a value greater than 1.0 indicating overcorrection and a value less than 1.0 indicating undercorrection. The CA is calculated by dividing TIA by SIA, that is, the inverse of the CI. This is the value used for the adjustment of the magnitude of future astigmatism treatments that is preferably 1. The FI is the dividing amount of astigmatism reduction achieved by the effective proportion of the SIA at the intended meridian by the TIA, which is preferably 1.0. Ocular residual astigmatism (ORA) is a dioptric measure of the noncorneal component of total refractive astigmatism, that is, the vector difference between refractive and corneal astigmatism [18].

### 2.4. Subgroup Analysis

Patients were categorized into three groups according to the axis of astigmatism: with-the-rule (WTR) astigmatism (steep corneal meridian from 60° to 120°); against-the-rule (ATR) astigmatism (steep corneal meridian either from 0° to 30° or from 150° to 180°); and oblique (OBL) astigmatism (all other astigmatism not belonging to WTR and ATR). Subgroup analyses were conducted to assess the magnitude of astigmatism and the change in astigmatism after surgery.

Patients were categorized into five groups according to their age: under 50 years; from 50 to 59 years; from 60 to 69 years; from 70 to 79 years; and 80 years or older. In addition, patients were categorized into three groups according to the longest WTW diameter: less than 11 mm; from 11 to 12 mm; and more than 12 mm. Subgroup analyses were also conducted to assess the correlation between the SIA and TIA magnitudes in each subgroup classified according to age and WTW.

### 2.5. Statistical Analyses

Data were presented as mean ± standard deviations. A Shapiro‒Wilk test was used to assess the distribution of the numerical data. Wilcoxon's signed-rank test was used to compare postoperative astigmatism with preoperative astigmatism. The Kruskal‒Wallis test was used to compare the magnitude of astigmatism or amount of astigmatic change after surgery between subgroups. Pearson's or Spearman's correlation analysis, depending on the distribution of data, was used to assess the relationship between the SIA and TIA magnitudes. Regression analysis was used to identify factors that affected the CI. Statistical significance was set at *P* < 0.05. All statistical analyses were performed using SPSS version 21.0 software (IBM SPSS Inc., IL, USA).

## 3. Results

Data were available for 79 eyes (56 right and 23 left eyes) of 79 patients who underwent routine examinations at one week, one month, and three months postoperatively. The mean age of these 35 males and 44 females was 66.95 ± 10.75 years.  Table 1 lists the preoperative patient demographics. All incisions were placed as intended, and no cases experienced inadvertent placement within the visual axis. There was no penetration of the Bowman or Descemet membranes, and all incisions were confined within the corneal stroma. No adverse events occurred during the follow-up period.

As a result of classifying subgroups according to the axis of astigmatism, 35 patients were categorized into the WTR group, 29 patients were categorized into the ATR group, and 15 patients were categorized into the OBL group. As a result of classifying subgroups according to the patient age, seven patients were under 50 years, seven patients were from 50 to 59 years, 28 patients were from 60 to 69 years, 30 patients were from 70 to 79 years, and seven patients were 80 years or older. As a result of classifying subgroups according to the longest WTW diameter, 12 patients were less than 11 mm, 58 patients were from 11 to 12 mm, and nine patients were more than 12 mm.

The values of corneal astigmatism measured by the autokeratometer and topography recorded preoperatively and at one and three months postoperatively are displayed in Table 2. We evaluated the values from the autokeratometer and scanning-slit topography and recorded all the data from the devices. Preoperative astigmatism was recorded as 1.23 ± 0.52 D from the autokeratometer and 1.17 ± 0.66 D from the scanning-slit topography. The corneal astigmatism decreased significantly at one and three months after surgery compared to the preoperative measurement, based on both devices (all *P* < 0.001).

**Figure 1:**
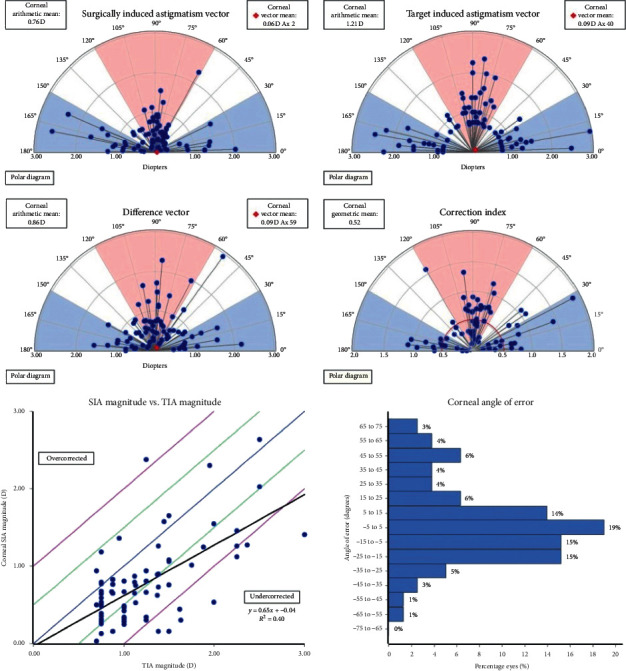
Outcomes of vector analysis by comparison of the preoperative with the postoperative (three months) values.

Table 3 and Figure 1 show the outcomes of vector analysis by the comparison of the preoperative with the postoperative (three months) values measured by the autokeratometer. The lower, left image in Figure 1 shows a statistically significant correlation between the overall SIA and TIA magnitudes. As a result of the linear regression, *r*^2^ = 0.40, *P* < 0.001, and overall CI was 0.62 ± 0.34 D, demonstrating undercorrection. As shown in the lower, right image in Figure 1, AE was in the highest proportion between −5° and 5°, and the average AE was 4.02° ± 28.7°. However, the absolute AE, which is the mean of the magnitude of AE, was 21.7° ± 19.0°.

**Figure 2:**
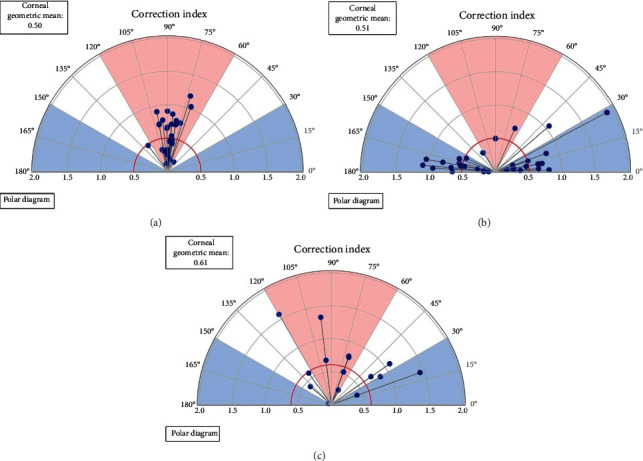
The single angle polar plots displaying the distribution of the correction index according to the astigmatism axis. (a) WTR, with-the-rule astigmatism; (b) ATR, against-the-rule astigmatism; and (c) OBL, oblique astigmatism.

The detailed values of the changes in these parameters measured by the autokeratometer according to the astigmatism axis are displayed in Table 4. The corneal astigmatism decreased significantly in all axes of astigmatism (*P*=0.001 or *P* < 0.001 in WTR and ATR; in OBL, *P*=0.013 at one month after surgery and *P*=0.040 at three months after surgery). The magnitudes of residual astigmatism were 0.89 ± 0.47 D in WTR, 0.75 ± 0.40 D in ATR, and 0.68 ± 0.46 D in OBL at three months after surgery. There was no significant difference between the subgroups in terms of either astigmatism magnitude or amount of reduction, regardless of the postoperative period (at one month after surgery, *P*=0.072 for the astigmatism magnitude and *P*=0.230 for the amount of reduction; at three months, *P*=0.217 for the astigmatism magnitude and *P*=0.639 for the amount of reduction).  Figure 2 depicts the single angle polar plots displaying the distribution of the correction index according to the astigmatism axis. The CI was 0.60, 0.58, and 0.77 in the WTR, ATR, and OBL subgroups, respectively.

**Figure 3:**
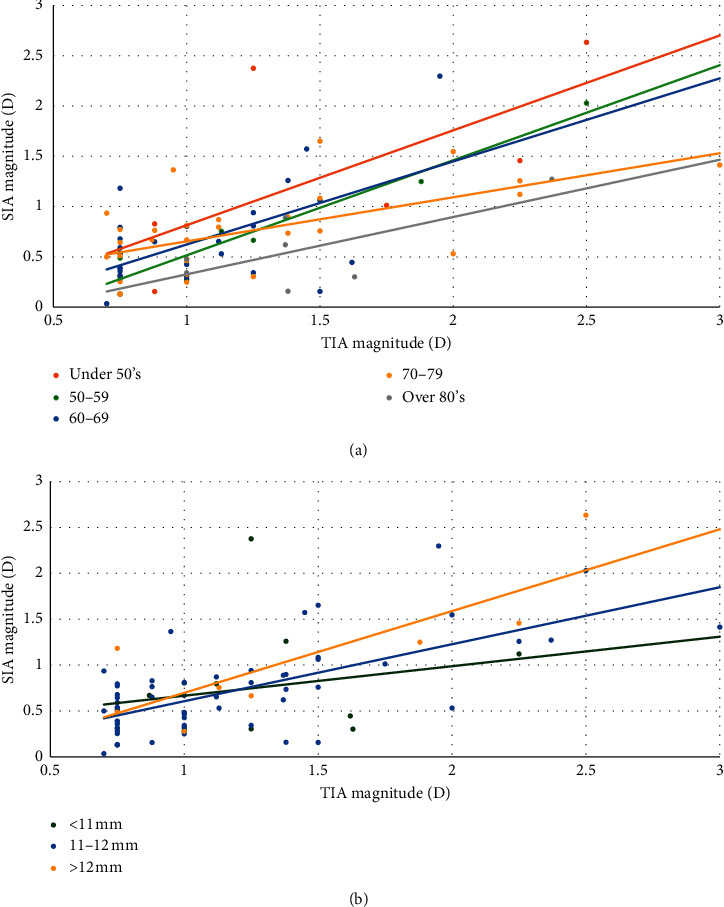
Correlation analysis between the surgically induced astigmatism and target-induced astigmatism in subgroups according to the patient age (a) and the longest white-to-white corneal diameter (b).

According to univariate regression analyses aimed at identifying factors that affected the CI at three months, there was a negative correlation between the age and the CI (*P*=0.022). The WTW, CCT, AE, and axis of astigmatism did not show any significant correlation with the CI. (*P*=0.810 for WTW, *P*=0.454 for CCT, *P*=0.654 for AE, *P*=0.182 for axis of astigmatism).  Figure 3 shows the correlation between the SIA and TIA magnitudes in the subgroups according to the age and the longest WTW. In the subgroup analysis according to age, a correlation between the TIA and SIA magnitudes was observed in all groups, except for those aged 80 years or older (*P*=0.021 for under 50 years, *P*=0.016 for 50 to 59 years, *P*=0.001 for 60 to 69 years, *P* < 0.001 for 70 to 79 years, and *P*=0.816 for 80 years or older). In correlation analysis in subgroup defined by WTW, a correlation between the TIA and SIA magnitudes was observed in all groups, except for those with the longest WTW less than 11 mm (*P*=0.473 for WTW less than 11 mm, *P* < 0.001 for WTW from 11 to 12 mm, and *P*=0.021 for WTW more than 12 mm).

## 4. Discussion

In this study, the combined FLACS and ISAK exhibited relatively satisfactory results but resulted in undercorrection of astigmatism, regardless of the meridian. In the univariate regression analysis of the factors affecting the CI, there was a significant negative correlation with age. There was no significant correlation with CI in terms of the type of astigmatism, CCT, or WTW.

In the present study, paired intrastromal keratotomy was performed, However, recently published studies have reported a lower CI in ISAK than in penetrating keratotomy. Day et al. used paired intrastromal keratotomies and reported a CI of 0.63 ± 0.32 after one month [19], while Byun et al., who used the same platform, reported a CI of 0.87 ± 0.50 after six months [20]. In contrast, Visco et al. reported a higher CI (0.94) at three months after paired penetrating keratotomy [11]. In the current study, which used paired intrastromal keratotomy, the CI was 0.62 ± 0.34 after three months, which was comparable to that reported in previous studies. Therefore, to improve the accuracy of ISAK, it is necessary to attempt to increase the lower CI, as compared to penetrating AK.

The astigmatism that could be corrected with AK varies depending on the study, but is generally limited to 3 D or less, and, in general, mild to moderate astigmatism is limited by a difficulty in the accurate measurement of the magnitude and axis of astigmatism. Thus, accurate evaluation of preoperative corneal astigmatism and consideration of various situations is essential for effective femtosecond-assisted ISAK. As the anterior corneal astigmatism shifts from WTR to ATR with aging while posterior corneal astigmatism remains as ATR, this should be considered and corrected [21]. In a penetrating AK-related study, the total corneal power and anterior astigmatism were significantly decreased after surgery, but posterior astigmatism remained unchanged [22]. Moreover, the ATR and OBL astigmatism had more marked image quality deterioration than WTR astigmatism [23]. In addition, various nomograms are currently used in femtosecond-assisted ISAK, although a perfect standard nomogram has not yet been developed, and there are many variations depending on the measurement devices of astigmatism. Thus, it is reasonable to recommend the postoperative target astigmatism as a WTR type from 0.25 to 0.5 D, rather than to strive for complete correction with 0 D [11, 24, 25].

The residual astigmatism measured by the autokeratometer at three months in this study was 0.80 ± 0.45 D. In previous studies, when ISAK was used, Day et al. reported 0.74 D at one month, Byun et al. reported 0.63 D at six months, and Chan et al. using penetrating AK reported 0.87 D at two months [19, 20, 26]. Residual astigmatism in this study was not larger than that of previous studies, and it could be expected that it would decrease slightly over the long period of time after surgery. According to a previous study using penetrating AK, the CI was 0.53, 1.01, and 0.95 in WTR, ATR, and OBL, respectively. Ideally, this value of CI is the result of undercorrection in WTR and full correction in ATR and OBL types [12]. When the nomogram of this study was applied, all groups exhibited a similar degree of undercorrection, which were appropriate for WTR, but was insufficient for ATR and OBL astigmatism as compared with the ideal amount of correction.

In the correlation analysis of factors that could affect CI, age alone showed a statistically significant negative correlation with CI. Currently used nomograms tend to decrease the arc length of ISAK in older patients. According to this result, it is necessary to increase the arc length to compensate for the lower CI in older patients, but aging-related changes in the cornea described above should be considered. Further research is needed on factors that can affect CI.

Based on the results of astigmatism correction in the nomogram we used in this study, the accuracy of our results could potentially be improved, and high CI could be achieved by the adjustment of the nomogram. For example, the arc length and axis of the nomogram could be adjusted using long-term CA or AE data or in consideration of the age-related change in astigmatism and CI as mentioned above. In a recently published study, a numeric planning tool using an optimization algorithm was used to reduce postoperative astigmatism with higher accuracy, which could be combined with our approach [27].

The current study was limited by its retrospective design, small sample size, and short follow-up period. In addition, among the preoperative corneal astigmatism values measured using various equipment, the inconsistent selection was used as the reference value for ISAK. In other words, in the autokeratometry, topography, partial coherence interferometry, and ocular aberrometry, slightly different astigmatism magnitudes and axes were measured. Among these, the surgeon determined the reference value of TIA as the most appropriate measurement value or an arbitrary value after considering several measurements. More reliable results could be expected in future studies that consistently apply one of the various measuring devices.

## 5. Conclusions

In conclusion, our study showed that FLACS and ISAK reduced postoperative corneal astigmatism effectively and safely. However, the precision of the nomogram should be improved through a long-term vector analysis on the results of AK and further research on the relationship between patient demographics and CI.

## Figures and Tables

**Table 1:** Preoperative patient characteristics and ocular biometric parameters.

*N* = 79	
Age	66.95, 10.75
Sex (M : F)	35 : 44
Laterality (OS : OD)	23 : 56
Astigmatism axis (ATR : WTR : OBL)	35 : 29 : 15
Preop endothelial cell count (cell/mm^2^)	2532.37, 424.47
Longest white-to-white (mm)	11.37, 0.50
Central corneal thickness (*μ*m)	585.35, 32.49

ATR, against-the-rule; WTR, with-the-rule; and OBL, oblique astigmatism. Data are given as numbers or as means, SD.

**Table 2:** Corneal astigmatism values recorded preoperatively and at 1 and 3 months postoperatively.

	Autokeratometer (KR-1®)			Topography (ORBscan®)		
	K Astig (mean, SD) range	*P*#	Δ† (mean, SD)	K Astig (mean, SD) range	*P*#	Δ† (mean, SD)
Preop	1.23, 0.52			1.17, 0.66		
Postop 1 mo	0.85, 0.42	<0.001	−0.38, 0.51	0.96, 0.45	<0.001	−0.21, 0.67
Postop 3 mo	0.80, 0.45	<0.001	−0.44, 0.55	0.94, 0.50	<0.001	−0.23, 0.73

ΔChange of corneal astigmatism compared with preoperative data. #Wilcoxon's signed-rank test (comparison with baseline time point). †Algebraic method.

**Table 3:** Outcomes of vector analysis via the comparison of the preoperative with the postoperative values measured by the autokeratometer.

	Mean, SD
TIA (D)	1.21, 0.52
SIA (D)	0.76, 0.53
DV (D)	0.86, 0.50
ME (D)	−0.46, 0.45
AE (degree)	4.02, 28.7
Absolute AE (degree)	21.7, 19.0
CI	0.62, 0.34
CA	2.48, 2.61
FI	0.45, 0.21
ORA (D)	0.06, 0.47

TIA, target-induced astigmatism; SIA, surgically induced astigmatism; DV, difference vector; ME, magnitude of error; AE, angle of error; Absolute AE, absolute angle of error; CI, correction index; CA, coefficient of adjustment; FI, flattening index; and ORA, ocular residual astigmatism.

**Table 4:** Detailed values of changes in these parameters measured by the autokeratometer according to the astigmatism axis.

		K Astig				
		K Astig (mean, SD)	*P* ^#^	Δ (mean, SD)	*P* ^*∗*^	*P* ^#^
Preop	WTR	1.34, 0.61	0.128			
	ATR	1.23, 0.46				
	OBL	1.00, 0.30				

1 month postop	WTR	1.00, 0.50	0.072	−0.34, 0.57	0.001	0.230
	ATR	0.75, 0.29		−0.48, 0.47	<0.001	
	OBL	0.71, 0.33		−0.29, 0.40	0.013	

3 months postop	WTR	0.89, 0.47	0.217	−0.45, 0.60	<0.001	0.639
	ATR	0.75, 0.40		−0.48, 0.48	<0.001	
	OBL	0.68, 0.46		−0.32, 0.54	0.040	

ATR, against-the-rule; WTR, with-the-rule; OBL, oblique astigmatism. ΔChange of corneal astigmatism compared with preoperative data. ^*∗*^Wilcoxon signed-rank test (comparison with baseline time point). ^*#*^Kruskal‒Wallis test between subgroups.

## Data Availability

The data used to support the findings of this study are available from the corresponding author upon request.
